# Classification of Schizophrenia, Bipolar Disorder and Major Depressive Disorder with Comorbid Traits and Deep Learning Algorithms

**DOI:** 10.21203/rs.3.rs-4001384/v1

**Published:** 2024-03-07

**Authors:** Xiangning Chen, Yimei Liu, Joan Cue, Mira Han Vishwajit Nimgaonkar, Daniel Weinberger, Shizhong Han, Zhongming Zhao, Jingchun Chen

**Affiliations:** The university of Texas Health Science Center at Houston; Director and CEO, Lieber Institute for Brain Development, Johns Hopkins School of Medicine: Departments of Psychiatry, Neurology, Neuroscience and Genetic Medicine; Director and CEO, Lieber Institute for Brain Development, Johns Hopkins School of Medicine: Departments of Psychiatry, Neurology, Neuroscience and Genetic Medicine; Director and CEO, Lieber Institute for Brain Development, Johns Hopkins School of Medicine: Departments of Psychiatry, Neurology, Neuroscience and Genetic Medicine; Director and CEO, Lieber Institute for Brain Development, Johns Hopkins School of Medicine: Departments of Psychiatry, Neurology, Neuroscience and Genetic Medicine; Lieber Institute for Brain Development; Johns Hopkins School of Medicine Department of Psychiatry and Behavioral Sciences; University of Texas HSC Houston

## Abstract

Recent GWASs have demonstrated that comorbid disorders share genetic liabilities. But whether and how these shared liabilities can be used for the classification and differentiation of comorbid disorders remains unclear. In this study, we use polygenic risk scores (PRSs) estimated from 42 comorbid traits and the deep neural networks (DNN) architecture to classify and differentiate schizophrenia (SCZ), bipolar disorder (BIP) and major depressive disorder (MDD). Multiple PRSs were obtained for individuals from the schizophrenia (SCZ) (cases = 6,317, controls = 7,240), bipolar disorder (BIP) (cases = 2,634, controls 4,425) and major depressive disorder (MDD) (cases = 1,704, controls = 3,357) datasets, and classification models were constructed with and without the inclusion of PRSs of the target (SCZ, BIP or MDD). Models with the inclusion of target PRSs performed well as expected. Surprisingly, we found that SCZ could be classified with only the PRSs from 35 comorbid traits (not including the target SCZ and directly related traits) (accuracy 0.760 ± 0.007, AUC 0.843 ± 0.005). Similar results were obtained for BIP (33 traits, accuracy 0.768 ± 0.007, AUC 0.848 ± 0.009), and MDD (36 traits, accuracy 0.794 ± 0.010, AUC 0.869 ± 0.004). Furthermore, these PRSs from comorbid traits alone could effectively differentiate unaffected controls, SCZ, BIP, and MDD patients (average categorical accuracy 0.861 ± 0.003, average AUC 0.961 ± 0.041). These results suggest that the shared liabilities from comorbid traits alone may be sufficient to classify SCZ, BIP and MDD. More importantly, these results imply that a data-driven and objective diagnosis and differentiation of SCZ, BIP and MDD may be feasible.

## Introduction

It is well known in psychiatry that comorbidities and overlapping phenomenology are common between different disorders, some symptoms are observed in multiple disorders, including physical diseases and behavioral traits.^1,2^ Over the years of genome wide association studies (GWASs), it has been clear that many of these comorbid disorders and traits share genetic liabilities.^3–6^ These shared liabilities suggest that not all genetic variants identified by a GWAS are specific to the disease, but it remains unclear to what extent that the liabilities shared with comorbid conditions account for the liability specific to the disease. From a clinical perspective, comorbidity increases the difficulties and challenges in disease diagnosis and treatment. Major psychiatric disorders such as schizophrenia (SCZ), bipolar disorder (BIP), and major depressive disorder (MDD) are often “misdiagnosed,” especially in the early stage of the disorders when typical symptoms have not been fully manifested. Understanding the genetic architecture of comorbid conditions could provide new insights into the underlying mechanisms and open new windows for a data driven, biology-based diagnosis and separation of these comorbid conditions.

**Figure 1:**
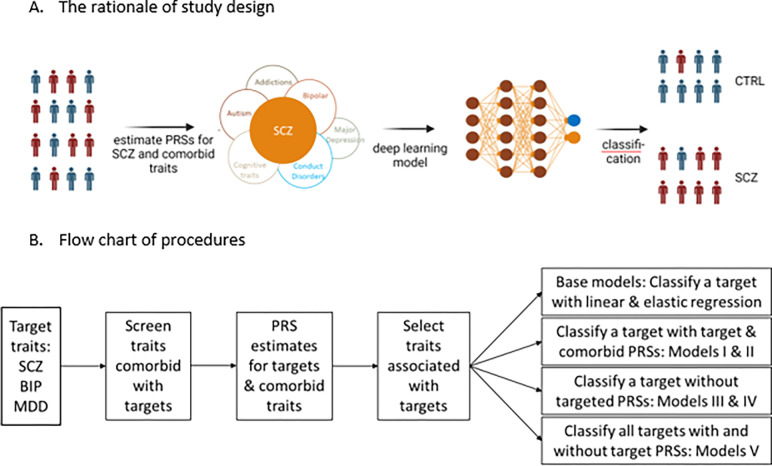
The rationale and study design. A. The rationale. Classification of a target trait, SCZ is used as an example. Conceptually, we can consider that the genetic liability of SCZ consists of a core of genetic factors that are specific to SCZ and a varying number of genetic factors from comorbid disorders and traits. The extent and variation of the sharing of genetic risks between individuals are explored with a deep neural network model to classify SCZ. B. A flow chart of procedures used in the study.

Conceptually, for an individual, we can consider that his/her genetic liability to a psychiatric disorder consists of a core of liability specific to the disorder and a peripheral shell made of shared liabilities from comorbid disorders and traits. For a given individual, the shared patterns and extents in the peripheral shell can be different from another individual. Therefore, we may be able to take advantage of these differences between people to separate affected individuals from unaffected individuals. Figure 1A illustrates this concept using SCZ as an example. Since polygenic risk score (PRS) has been established as a reliable approximation of genetic liability,^7,8^ we can consider that the total genetic liability of a disorder for a given individual is the sum of PRS of the targeted disorder and the PRSs of all other comorbid disorders and traits. Similarly, we can extend the concept of overlapping genetic liabilities to several disorders with common clinical symptoms. Due to the differences in the degree of overlaps among these disorders, we can utilize these differences to distinguish major psychiatric disorders that have substantial overlaps in both genetic liabilities and clinical symptoms. Based on these rationales, for a specific disorder, we can construct a classification model that integrates the PRS of targeted disorder with the PRSs of all other comorbid disorders and traits, and this model may have a better performance than the model that uses the PRS of the targeted disorder alone. Similarly, we can also build models with these PRSs to differentiate several different but symptomatically overlapped disorders.

We have performed this study as a demonstration of these principles. We searched diseases and traits that are comorbid with SCZ, BIP, and MDD from the literature, and matched these traits with those reported in the GWAS catalog ^9,10^ (https://www.ebi.ac.uk/gwas/). We then calculated the PRSs for SCZ, BIP, MDD, and the comorbid traits, and evaluated their genetic correlations. We selected those traits with PRSs statistically associated with the targeted disorders (SCZ, BIP, and MDD), and constructed deep neural networks (DNN) models with these PRSs to evaluate their utilities in the classification and differentiation of the targeted disorders (Figure 1B). The results obtained from the models could help us understand the genetic architecture of comorbid conditions and provide strategies for biology-based diagnosis and distinction of these comorbid conditions.

## METHODS

### 3.1 Datasets and genotype imputation

In this study, we used datasets obtained from multiple sources. For SCZ datasets, we used the Molecular Genetics of Schizophrenia (MGS) ^11^ (accession phs000167.v1.p1, cases = 2,681, controls = 2,653) and the Swedish Case-Control Study of Schizophrenia (SCCSS) ^12^ ( accession phs000473.v2.p2, cases = 2,895, controls = 3,836) from dbGaP (https://www.ncbi.nlm.nih.gov/gap/), and the Clinical Antipsychotic Trials of Intervention Effectiveness ^13,14^ (CATIE, cases = 741, controls =751) from NIMH’s genetic repository (https://www.nimhgenetics.org/). These SCZ datasets were combined with PLINK software.^15,16^ For BIP datasets, we used samples from the Psychiatric Genomics Consortium (PGC) (TOP3, cases = 203, controls = 349; DUB, cases = 150, controls = 797; EDI, cases = 282 controls = 275) and the Wellcome Trust Case Control Consortium (https://www.wtccc.org.uk/) (cases = 1998, controls = 3004). The BIP datasets were also combined as a single sample using PLINK. The MDD data was obtained from dbGaP, accession phs000486.v1.p1, with 1,704 cases and 3,357 controls.^17,18^ For all datasets, we obtained the genotype and phenotype information from the sources, conducted genotype quality assessments, and removed SNPs with minor frequency less than 0.01 and Hardy-Weinberg equilibrium p-value less than 0.0001. We then conducted genotype imputations for each dataset separately using the Michigan Imputation Server (https://imputationserver.sph.umich.edu/index.html#!) using the 1000 Genomes Phase 3 as reference and default parameters. After the imputation, SNPs with INFO value less than 0.4 were removed. For analyses that required combining the datasets (i.e., main models V), we used PLINK to merge the datasets. For phenotypes, we used the same definitions as defined in the original studies.

### 3.2 Selection of comorbid traits and PRS calculation

We reviewed literature on comorbid traits of psychiatric disorders and searched the GWAS catalog^9,10^to find whether the traits had GWAS performed. If a GWAS was found, the summary statistics would be downloaded. Of note, a trait here was defined as a phenotype that GWAS was performed for. For a psychiatric disorder, if multiple related phenotypes were used for GWAS, all of these phenotypes were considered individual traits and were included in our study. With the summary statistics of GWASs, we used the default settings of PRSice2 package ^19,20^to calculate the PRSs for the best-fit p-value threshold (bestPRS, hereafter) for the SCZ, BIP, and MDD samples for each candidate trait. For a candidate trait, if the association p-value of the bestPRS with any one of our targeted disorders was ≤ 0.050, we would include it in our study. With this procedure, a total of 42 traits were obtained (supplementary Table S1). For all included traits, we then calculated PRSs at six p-value thresholds (5e-8, 1e-5, 1e-3, 0.1, 1 and the best-fit p-value) for all subjects of the datasets used in this study. For all PRSs, we rescaled them to the range between 0 and 1 and stored them as individual by feature matrices for model inputs.

### 3.3 Model definition, training, and optimization

We used 6 main models in this study. For convenience, we used this convention to name our models: main_model.submodel.target_trait. If the main models did not have submodels, then they would be named as main_model.target_trait. The details of models were listed in Table 1. We used logistic and elastic regressions to establish a baseline (main model B) to compare to DNN models. For the elastic models, we used a grid search to find the optimal alpha value and L1 ratio (alpha of 0.010 and L1 ratio of 0.920) for submodels B.II and B.III.

Main models I to IV were binary models designed to classify the target disorders using various PRS combinations. Main model V was a multiclass model, and we used it to classify the 4 classes of CTRL, SCZ, BIP and MDD by combining the datasets from the 3 targeted diseases together. To account for batch effects among the 3 datasets, we conducted batch correction with the pyComBat package.^21,22^ Since the number of subjects in each class was substantially different, the combined dataset from the 3 diseases was imbalanced for the 4 classes, therefore, we used oversampling techniques (ADASYN ^23^ and borderline SMOTE ^24,25^) to balance the classes and train the models.

For models that needed to remove targeted disease and related phenotypes, i.e., models B.III, III, IV, V.III, and V.IV (see model definitions below), PRSs obtained for these phenotypes would be removed from the models (see supplementary Table S1, columns 5–8). For models that used multiple levels of PRSs for the same traits, i.e., models III and V.III, when a trait was removed from the model, all levels of PRS of that trait would be removed.

W used the TensorFlow (version 2.5.0; www.tensorflow.org/),^26,27^ keras (version 2.9.0; https://keras.io/api/) and DNN architecture to construct the models. An example of the models (model II.SCZ) was shown in supplementary Figure S1, and the Python scripts for main models could be found in our Github site (https://github.com/mdsamchen/scz_bip_mdd). For each model, we used the leave one out cross validation (LOOCV) procedure to conduct validation. All results reported were obtained from the 20% left-out samples that were not seen by any of the models during the training processes. For binary classification (main models B, I to IV), we reported the binary accuracy, precision, recall, F1 score, and AUC as defined in the scikit-learn package (version 0.23.2).^28^ For multiclass model (i.e., model V), we reported the average of categorical accuracies from all classes, and the AUCs were also averaged across the classes. The precision, recall, and F1 score were reported for each class.

### 3.4 Evaluation of feature importance

For DL models, permutations had been used as a method to evaluate the importance of the features.^29^ We use permutations to estimate the importance of the features by the following procedures: a). define the feature importance as the change of model performance. For binary classification models, we used r2 score, as defined in the scikit-learn package, as the measurement of model performance. For multiclass classification models, we used class weighted average of AUC as the measurement. To estimate the feature importance, we permuted each feature 100 times for the trained model and took the average of these 100 permutations as the performance of the permuted feature. The importance of the feature was the difference between the performance of the trained model and the permuted feature:

$$i_{j}=s-\frac{1}{K}\sum_{k=1}^{K}\;s_{k,j}$$

Where $i_{j}$ is the importance of feature j,s is the performance score (r2 score for binary model and AUC for multiclass model) of the trained model, K is the number of permutations. We used one sample t-test to evaluate whether the change was statistically significant assuming that the performance changes from permutations followed normal distribution. The changes of the permuted performances were plotted using Seaborn (version 0.12.2) ^30^ and Matplot (version 3.5.2; https://www.mathworks.com/help/stats/index.html) libraries.

### 3.5 UMAP plotting of model embeddings

Model embeddings were extracted from the layer immediately before the classification layer. For direct comparison between the models with and without the inclusion of target specific PRSs, all models had 32 dimensions at this embedding layer. Then the embeddings were projected into a 2-dimensional space by umap-learn (version 0.5.4) ^31^ and plotted by classes with the Matplot library.

## RESULTS

### 4.1 Selection of comorbid traits

Based on the survey of the literature ^3,32–35^ and our test of association, we selected a total of 42 diseases/traits in this study, including the targeted disorders (see Supplementary Table S1). From Table S1, it was clear that the associations between the targeted disorders and comorbid PRSs vary substantially. All selected traits are associated with at least one of our targeted disorders, i.e., SCZ, BIP, or MDD.

### 4.2 Comparison between baseline models with and without the inclusion of PRSs from target disorder and comorbid traits

**Figure 2:**
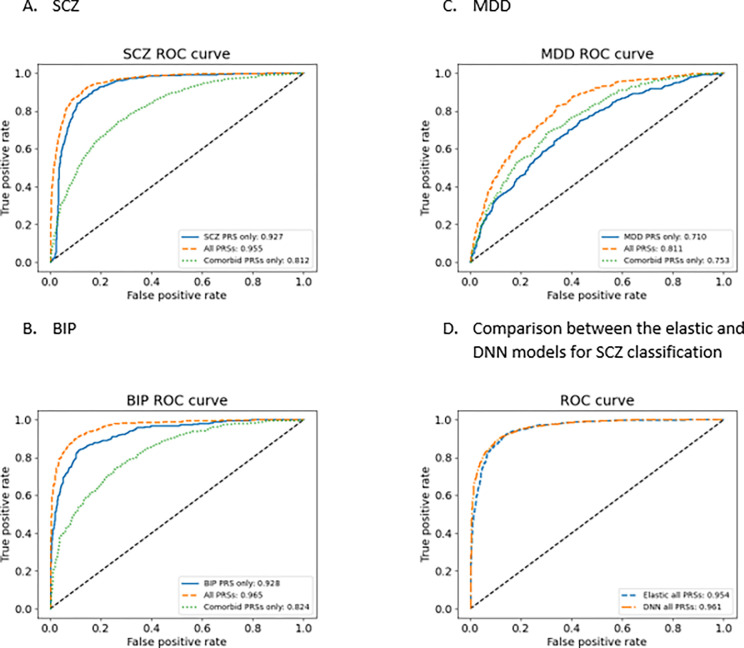
Base model performances for SCZ (A), BIP (B) and MDD (C). The plots show the ROC curves for the baseline models that use only the bestPRSs from the targeted disorders (blue, Model B.I), bestPRSs from all traits (targeted disorders and comorbid traits, gold, Model B.II), and bestPRSs from only the comorbid traits (green, Model B.III). The results indicate that models with the inclusion of PRSs from comorbid traits have better performances than that of using only the PRSs from targeted traits. It is also clear that models using only the PRSs from comorbid traits can also have decent performance. Panel D is a comparison between the baseline elastic model (Model B.II.SCZ) and DNN for SCZ classification (Model II.SCZ).

We started our study by building models using the bestPRSs and sex to classify targeted disorders and treated them as the baseline models for comparison with all other models. For each targeted disorder, there were 3 baseline models, B.I, B.II, and B.III, which used targeted bestPRS, all best PRSs, and all bestPRSs but targeted bestPRS as predictors respectively (Table 1). The performances of these baseline models for SCZ were summarized in Tables S2. Inclusion of PRSs from comorbid traits did improve model performance (AUC with about 2.8% improvement) (Figure 2A, Table S2), but the improvement was modest. Similar improvements were also observed in class specific precision, recall, and F1-score (Table S2, models B.I.SCZ and B.II.SCZ). Model B.III used only the PRSs from comorbid traits, and surprisingly, model B.III.SCZ achieved an accuracy of 0.734 ± 0.001 and AUC of 0.812 ± 0.001. While the performance of model B.III.SCZ was worse (Figure 2A, Table S2) than that of models B.I.SCZ and B.II.SCZ, the results were intriguing given the fact that no bestPRSs of SCZ and directly related traits were included in model B.III.SCZ.

For BIP, we observed a similar trend as observed in SCZ, i.e., model B.II.BIP performance > model B.I.BIP performance > model B.III.BIP performance (Figure 2B, Table S3). But for MDD, model B.III.MDD had a better performance than model B.I.MDD (Figure 2C, Table S4). Overall, these results indicated that inclusion of PRSs from comorbid traits improved model performances, and the use of only the PRSs of comorbid traits could predict disease status for SCZ, BIP, and MDD.

We evaluated the performances between the elastic regression models and DNN models using the bestPRS dataset of SCZ (comparison between models B.II.SCZ and II.SCZ), and the two models performed virtually the same (Figure 2D).

### 4.3 Classification of SCZ with DNN models

We constructed 4 DNN models to classify SCZ diagnosis with the PRSs obtained from the selected traits. For all models, sex was included as a predictor. For model I.SCZ, using a 5-fold LOOCV scheme, we obtained accuracy and AUC for the left-out samples of 0.913 ± 0.004 and 0.974 ± 0.002, respectively. The class specific precision, recall, and F1-score for SCZ were 0.915 ± 0.004, 0.912 ± 0.005, and 0.913 ± 0.005, respectively (Table 2, model I.SCZ). Please note that while there were some differences between SCZ and CTRL for class specific precision, recall, and F1-score matrix, the results for the two classes were comparable.

For model II.SCZ, we built a DNN model (Figure S1) and used the same LOOCV procedures for model training and validation. The performance was very close to that of model I.SCZ, the accuracy and AUC were 0.880 ± 0.005 and 0.956 ± 0.003, respectively (Table 2, model II.SCZ). The SCZ specific precision, recall, and F1-score were close to those of model I.SCZ as well. Of note, model II.SCZ (Table 2) and model B.II.SCZ (Table S2) used exactly the same predictors, the two models performed virtually the same for accuracy, AUC, and class specific precision, recall, and F1-score.

For model III.SCZ, after removing all PRSs of the traits that included SCZ phenotypes directly in their prospective GWASs, we were surprised to find that the model performed reasonably well, with a validation accuracy of 0.760 ± 0.007 and a validation AUC of 0.843 ± 0.005 (Table 2, model III.SCZ). The SCZ specific precision, recall, and F1-score were 0.784 ± 0.009, 0.719 ± 0.023, and 0.749 ± 0.012. Although the accuracy and AUC, and the class specific matrices were substantially lower than that of model I.SCZ (Table 2, comparing models I.SCZ and III.SCZ), the results remained significant.

For model IV.SCZ, the accuracy and AUC were significantly lower than that of model II.SCZ, but the results were decent, with validation accuracy of 0.710 ± 0.008 and validation AUC of 0.789 ± 0.011 (Table 2, model IV.SCZ vs. model II.SCZ). Similar trends were also observed for the class specific matrices.

### 4.4 Classification of BIP and MDD with DNN models

We pursued similar strategies for the classification of BIP and MDD with DNN models. The results for BIP were summarized in Table 3, which closely mirrored those of SCZ reported in Table 2 except that models III.BIP and IV.BIP had virtually the same performance.

For MDD, while there were some differences among the 4 models, the performances were generally on a par with one another, including class specific matrices (Table 4). This was different from the trend observed from both SCZ and BIP. Additionally, the overall performance of MDD models was worse than that of SCZ and BIP. This could be due to the difference in heritability between these disorders.

### 4.5 Classification of control, SCZ, BIP, and MDD

**Figure 3:**
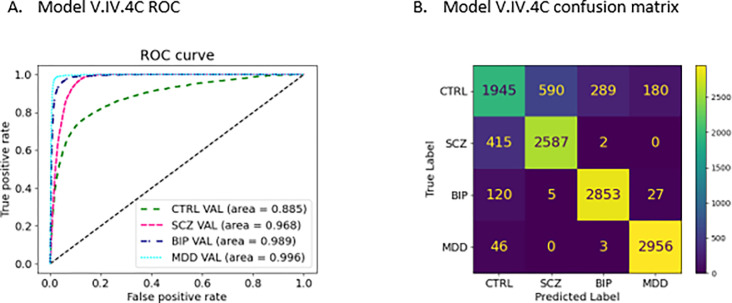
The ROCs and Confusion matrices for Models V. The worst performance (A), and the confusion matrix showed that misclassifications largely came from the CTRL group (B), the classification of the other 3 groups were good.

We built a multiclass model, model V, to classify and differentiate the 4 classes (CTRL, SCZ, BIP and MDD). The results were summarized in Table 5. Among the models, model V.I.4C had a similar performance as model V.II.4C and model V.III.4C had a similar performance as model V.IV.4C. For example, the average AUC of model V.I.4C (0.986 ± 0.015) was very close to that of model V.II.4C (0.990 ± 0.010), and so forth (Table 4). As expected, models with the inclusion of PRSs from targeted diseases, i.e., models V.I.4C and V.II.4C, had better performances than that of models without PRSs from targeted diseases (models V.III.4C and V.IV.4C). For all models, the class specific measures for the CTRL were the worst (Table 5, Figure 3A). When we examined the confusion matrices more carefully, it was apparent that most of the misclassifications occurred with the controls (Figure 3B).

### 4.6 Evaluation of contribution of comorbid traits to SCZ, BIP and MDD

**Figure 4:**
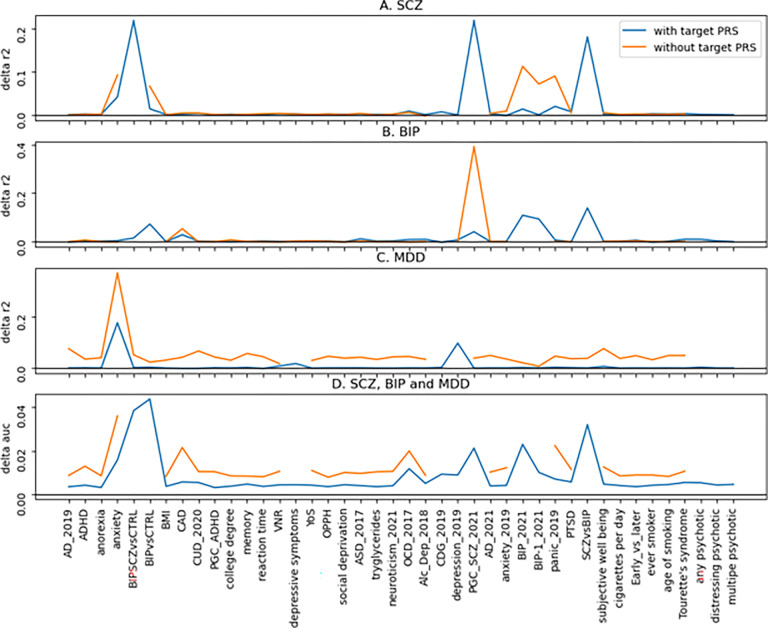
Comparison of feature importance between models with and without the inclusion of the targeted PRSs. Permutation based feature importance estimates, as measured by delta R2 scores and delta AUC, were plotted for the two models. The features not included in the models were replaced with NA, and plotted as broken lines. A. SCZ, models II.SCZ and IV.SCZ. B. BIP, models II.BIP and IV.BIP. C. MDD, models II.MDD and IV.MDD. D. SCZ, BIP, and MDD, models V.II.4C and V.IV.4C.

**Figure 5:**
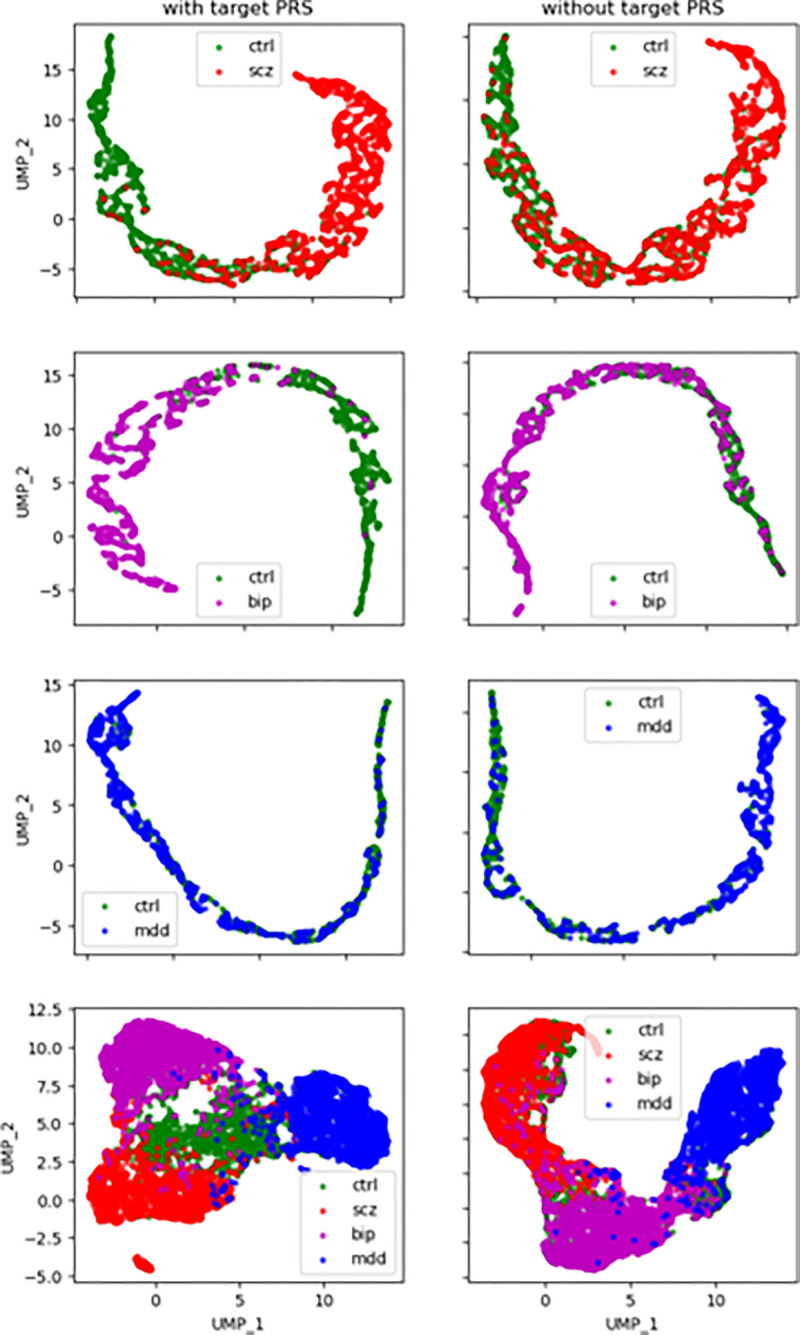
Comparisons of the embedding structures between models with and without the inclusion of targeted PRSs. The embedding layers immediately before the classification layer of the models were extracted and projected into a 2-dimension space using the UMAP method. Panels on the left side were from models with the inclusion of targeted PRSs (II.SCZ, II.BIP, II.MDD and V.II.4C), and panels on the right side were from models without the inclusion of targeted PRSs (IV.SCZ, IV.BIP, IV.MDD and V.IV.4C). For binary classification models, there were no apparent differences in cluster structures. For the multiclass models, model V.IV.4C did not have a CTRL cluster (green), and BIP became the connecting cluster between the SCZ and MDD clusters.

Based on the results from the models that did not include the PRSs from the target and directly related traits, PRSs from comorbid traits could substitute for the target PRSs, but we did not know what comorbid traits were involved. To answer this question, we implemented a permutation procedure to evaluate the importance of the features in the models. Figure 4 shows the results as measured by the changes of r2 score (delta r2) and AUC (delta auc) between models II and IV for SCZ, BIP and MDD, and between models V.II.4C and V.IV.4C. From the first panel, the SCZ panel, for model II.SCZ, 3 predictors/traits, i.e., BIP and SCZ vs CTRL, PGC_SCZ_2021, and SCZ vs BIP, made the most contribution to the model (Figure 5A). When these traits were removed from the model, i.e., model IV.SCZ, anxiety, BIP versus CTRL, BIP_2021, BIP-1_2021 and panic_2019 became the most important contributors of the model. In other words, these 5 traits could largely account for the effects of SCZ specific traits in model II.SCZ. Similarly, for BIP and MDD (Figure 4B and 4C), PGC_SCZ_2021 and anxiety could largely account for the effects of BIP- and MDD-specific traits respectively. For the multiclass classification model V.II.4C, in addition to those traits directly involved in SCZ, BIP and MDD (BIP and SCZ vs CTRL, BIP vs CTRL, depression_2019, PGC_SCZ_2021, BIP_2021 and SCZ vs BIP), anxiety and OCD_2017 also made substantial contributions to the model. After removing target specific PRSs, anxiety, CAD (coronary artery disease), OCD_2017, anxiety_2019 and panic_2019 became the major players in model V.IV.4C. Intriguingly, a physical disease, coronary artery disease, became a prominent contributor in the classification and differentiation of CTRL, SCZ, BIP and MDD.

We also examined the embedding projections of these models to see whether there were significant differences in cluster structures. The results are presented in Figure 5. For the binary classification models, with or without the use of target specific PRSs, the models had similar embedding structures. For example, for models II.SCZ and IV.SCZ, shown as the top two panels in Figure 5, while there were significant differences in model performance, i.e., classification accuracy, there was no apparent difference in cluster structure and were no apparent gaps in embedding projections. The difference between models V.II.4C and V.IV.4C, the multiclass classification models, was the disappearance of the CTRL group, and BIP became the group connecting the SCZ and MDD groups.

## DISCUSSION

In this study, we used PRSs from multiple comorbid traits to classify SCZ, BIP and MDD. Our study shows that PRSs from both target traits and comorbid traits can consistently predict the disease status of the targets. The results from models I and II might be inflated because the samples we used are part of the GWASs for SCZ, BIP, and MDD. The original reason we designed models III and IV was to address the sample independence issue. We reasoned that if we excluded all PRSs obtained from the targeted disorder and directly related phenotypes, we could establish the lower bound of the model performance. The results we observed exceeded our expectations and were exciting. Based on our reading of the literature, there is no report that PRSs from comorbid traits alone can classify major psychiatric disorders, such as SCZ, BIP, and MDD. Although we ^36^ and others ^37,38^ have reported that inclusion of comorbid traits could improve the classification of targeted disorders, but there are no reports of classification that exclude the PRSs of targeted disorders.

Our study has two major findings. One is that the PRSs from the comorbid traits alone, i.e., without the inclusion of PRSs of targeted diseases, can classify the targeted diseases, and these models perform reasonably well. This observation is true for the 3 psychiatric disorders studied in this article. This finding is consistent with recent reports that major psychiatric disorders share significant genetic liabilities,^3,4,39^ suggesting that many of the risk alleles found in disorder specific GWASs may be the same alleles found in a different GWAS. Our study explicitly shows that a combination of PRSs from comorbid traits can replace the target specific PRSs to predict the disease status of the targets, and we can quantify the effects of target specific risks by comparing the performance between models II and IV. Furthermore, by examining the feature importance of these models, we can find which traits can be used to replace the targets in perspective models. In the case of BIP, PRSs from coronary artery disease (CAD) and SCZ can substitute the BIP specific PRSs (Figure 4B).

This finding raises an interesting question, that is how many genetic risk variants found in a GWAS are specific to the target disease, say, SCZ? In this study, we used a total of 42 traits, and the 35 traits not directly related to SCZ could predict SCZ status reasonably well. From the feature importance analyses, anxiety, BIP and panic can largely compensate the effects of SCZ specific PRSs (Figure 4A). The differences in model performance between model II.SCZ and IV.SCZ were 0.170 (accuracy) and 0.147 (AUC) (Table 2). The differences between II.BIP and IV.BIP and between II.MDD and IV.MDD were even smaller. Since the comorbid traits used in our models were far from exhaustive, other potential traits could be included. For examples, breast cancer,^40^ migraine^41^ and amyotrophic lateral sclerosis^42^ have been reported to have genetic correlation with SCZ, they could be good candidates to expand our list of comorbid traits. Should more genetically correlated traits be included, we would reasonably expect that the gap between models II.SCZ and IV.SCZ would decrease, i.e., the number of variants specific to SCZ would decrease, and this observation can be extended to BIP and MDD as well. In other words, the risk variants specific to SCZ, BIP or MDD are limited, most variants found in disease specific GWAS are shared between comorbid traits. The consequence is that, if we believe that the main roles of genetic risks on SCZ are disruption of normal biological process/functions and our finding that many, perhaps, a majority, of the genetic risks are not specific to SCZ, it would lead to a conclusion that unless we know that a drug is designed to target variants specific to SCZ, the drug is unlikely to have a specific effect to SCZ, and it is more likely to be interchangeable among multiple disorders. This is consistent with current clinical practice that several drugs are exchangeable in treating SCZ, BIP and MDD because they all target the same or similar mechanisms.^43^

Another potentially important finding is that with PRSs from multiple comorbid traits, we can effectively differentiate CTRL, SCZ, BIP, and MDD. The results remained significant with or without the inclusion of the PRSs of targeted diseases (Tables 5). When we examine the results more carefully, we find that the CTRL group has the lowest AUC and significant misclassifications (Fig. 3A and 3B), leading to lower precision, recall and F1-score for this group. A possible reason could be that the controls used in the datasets were not likely super controls who did not have any symptoms or diagnoses for all comorbid traits included in this study. The PRSs from comorbid traits help to differentiate each patient group (SCZ, BIP, or MDD), but they may blur the line between controls and case groups (SCZ, BIP, or MDD). This is because controls in the SCZ, BIP, and MDD datasets might not have been screened against all the comorbid traits, such as years of school attended ,^44^ body mass index,^45^ and memory and neural function measures,^46^ traits for which GWAS based PRSs were used in the models.

Our study has some limitations. One is potential overlap of controls between the targeted GWASs (i.e., SCZ, BIP, and MDD) and the GWASs of the traits included in our study. Some GWASs use consortium data that include control subjects from multiple sources. While these overlapping control subjects may not impact these separate GWASs, it might lead to some dependences between our targeted disorders and those comorbid traits if their GWASs share some control subjects. Since our study used a substantial number of GWASs, it was difficult for us to know whether and to what extent these GWASs have overlapping controls. Therefore, it would be difficult to estimate their impact on our model’s performance. Independent studies may be needed to validate our findings. Another potential issue is the differentiation of the targeted disorders. The samples for SCZ, BIP, and MDD studies come from different sources, even though we conducted batch effect correction before using the data in our model, it may not be sufficient to account for the stratification, leading to inflated performance. Further studies with the same genotype platform and samples coming from the same populations are necessary to validate our findings.

In summary, we find that without the use of PRSs of targeted disorders, we can predict the status of these disorders. These results suggest that most genetic risk variants found by GWASs may not be specific to the disorders. Furthermore, PRSs from comorbid conditions, with or without the inclusion of PRSs from targeted disorders, can be used to differentiate the targeted disorders, indicating that it may be feasible to obtain a data-driven and biology-based diagnosis for these disorders.

## Figures and Tables

**Table 1.** Model information*

Main Model	Submodel	Target	Structure	Traits (# Predictors)
Baseline	I	SCZ; BIP; MDD	logistic regression	target bestPRS (1)
Baseline	II	SCZ; BIP; MDD	elastic regression	all bestPRSs (42)
Baseline	III	SCZ; BIP; MDD	elastic regression	exclusion of target bestPRSs, SCZ (35); BIP (33); MDD (36)
I		SCZ; BIP; MDD	deep neural network	all PRSs, SCZ (237); BIP (237); MDD (237)
II		SCZ; BIP; MDD	deep neural network	all bestPRSs, SCZ (42), BIP (42); MDD (42)
III		SCZ; BIP; MDD	deep neural network	exclusion of target PRSs, SCZ (196); BIP (184); MDD (202)
IV		SCZ; BIP; MDD	deep neural network	exclusion of target bestPRSs, SCZ (35); BIP (33); MDD (36)
V	I	CTRL, SCZ, BIP and MDD	deep neural network	all PRSs (237)
V	II	CTRL, SCZ, BIP and MDD	deep neural network	all bestPRSs (42)
V	III	CTRL, SCZ, BIP and MDD	deep neural network	exclusion of target PRSs (166)
V	IV	CTRL, SCZ, BIP and MDD	deep neural network	exclusion of target bestPRSs (30)

*: Model nomenclature: Main_model.submodel.target_disorder. If a main model does not have a submodel, then main_model.target_disorder. For example, B.I.SCZ, I.SCZ and V.I.4C.

**Table 2.** Classification of schizophrenia

Model	Class	Accuracy	AUC	Precision	Recall	F1-score
I.SCZ	CTRL	0.913 ± 0.004	0.974 ± 0.002	0.912 ± 0.005	0.915 ± 0.004	0.914 ± 0.004
	SCZ			0.915 ± 0.004	0.912 ± 0.005	0.913 ± 0.005
II.SCZ	CTRL	0.880 ± 0.005	0.956 ± 0.003	0.884 ± 0.004	0.875 ± 0.009	0.879 ± 0.006
	SCZ			0.876 ± 0.008	0.885 ± 0.003	0.881 ± 0.006
III.SCZ	CTRL	0.760 ± 0.007	0.843 ± 0.005	0.740 ± 0.014	0.802 ± 0.014	0.770 ± 0.006
	SCZ			0.784 ± 0.009	0.719 ± 0.023	0.749 ± 0.012
IV.SCZ	CTRL	0.710 ± 0.008	0.789 ± 0.011	0.713 ± 0.014	0.702 ± 0.005	0.707 ± 0.007
	SCZ			0.706 ± 0.005	0.718 ± 0.020	0.712 ± 0.012

**Table 3.** Classification of bipolar disorder

Model	Class	Accuracy	AUC	Precision	Recall	F1-score
I.BIP	CTRL	0.895 ± 0.020	0.965 ± 0.003	0.904 ± 0.066	0.892 ± 0.056	0.894 ± 0.018
	BIP			0.896 ± 0.040	0.900 ± 0.084	0.894 ± 0.031
II.BIP	CTRL	0.904 ± 0.014	0.965 ± 0.001	0.924 ± 0.027	0.883 ± 0.056	0.901 ± 0.021
	BIP			0.890 ± 0.042	0.925 ± 0.030	0.906 ± 0.012
III.BIP	CTRL	0.768 ± 0.007	0.848 ± 0.009	0.760 ± 0.013	0.782 ± 0.016	0.771 ± 0.007
	BIP			0.775 ± 0.010	0.752 ± 0.021	0.764 ± 0.010
IV.BIP	CTRL	0.782 ± 0.006	0.852 ± 0.004	0.787 ± 0.005	0.770 ± 0.011	0.778 ± 0.008
	BIP			0.775 ± 0.009	0.792 ± 0.004	0.783 ± 0.006

**Table 4.** Classification of major depressive disorder

Model	Class	Accuracy	AUC	Precision	Recall	F1-score
I.MDD	CTRL	0.782 ± 0.015	0.854 ± 0.010	0.784 ± 0.046	0.785 ± 0.053	0.782 ± 0.007
	MDD			0.787 ± 0.029	0.778 ± 0.083	0.779 ± 0.034
II.MDD	CTRL	0.782 ± 0.004	0.848 ± 0.007	0.794 ± 0.020	0.763 ± 0.030	0.778 ± 0.007
	MDD			0.773 ± 0.017	0.801 ± 0.033	0.786 ± 0.010
III.MDD	CTRL	0.794 ± 0.010	0.869 ± 0.004	0.800 ± 0.017	0.767 ± 0.025	0.783 ± 0.016
	MDD			0.783 ± 0.018	0.813 ± 0.021	0.797 ± 0.013
IV.MDD	CTRL	0.753 ± 0.019	0.822 ± 0.010	0.739 ± 0.047	0.793 ± 0.057	0.762 ± 0.011
	MDD			0.779 ± 0.031	0.712 ± 0.093	0.740 ± 0.043

**Table 5.** Classification of controls and patients diagnosed with schizophrenia, bipolar and major depression

Model	Class	Accuracy*	AUC**	Precision	Recall	F1-score
V.I.4C	CTRL	0.911 ± 0.009	0.986 ± 0.015	0.856 ± 0.076	0.821 ± 0.065	0.834 ± 0.008
	SCZ			0.938 ± 0.025	0.942 ± 0.044	0.939 ± 0.012
	BIP			0.954 ± 0.022	0.932 ± 0.054	0.941 ± 0.023
	MDD			0.907 ± 0.026	0.948 ± 0.041	0.926 ± 0.009
V.M.4C	CTRL	0.938 ± 0.003	0.990 ± 0.010	0.914 ± 0.024	0.841 ± 0.029	0.875 ± 0.008
	SCZ			0.942 ± 0.007	0.962 ± 0.013	0.952 ± 0.003
	BIP			0.959 ± 0.015	0.972 ± 0.010	0.965 ± 0.004
	MDD			0.936 ± 0.015	0.977 ± 0.006	0.956 ± 0.005
V.NI.4C	CTRL	0.850 ± 0.017	0.960 ± 0.041	0.733 ± 0.064	0.659 ± 0.046	0.690 ± 0.016
	SCZ			0.823 ± 0.013	0.857 ± 0.057	0.838 ± 0.023
	BIP			0.902 ± 0.011	0.927 ± 0.039	0.914 ± 0.016
	MDD			0.938 ± 0.006	0.956 ± 0.018	0.947 ± 0.010
V.IV.4C	CTRL	0.861 ± 0.003	0.961 ± 0.041	0.768 ± 0.013	0.655 ± 0.029	0.706 ± 0.013
	SCZ			0.825 ± 0.009	0.868 ± 0.020	0.846 ± 0.007
	BIP			0.893 ± 0.011	0.944 ± 0.004	0.918 ± 0.005
	MDD			0.941 ± 0.008	0.976 ± 0.005	0.959 ± 0.003

*: average categorical accuracy from all classes

**: average AUC from all classes
